# Targeted metabolomic profiles of serum amino acids are independently correlated with malnutrition in older adults

**DOI:** 10.1186/s12877-024-04937-y

**Published:** 2024-04-15

**Authors:** Xianghui Zhao, Li Meng, Daguang Wang, Jing Shi, Wenbin Wu, Guoqing Fan, Hong Shi, Jun Dong, Pulin Yu, Ruiyue Yang

**Affiliations:** 1 The Key Laboratory of Geriatrics, Beijing Institute of Geriatrics, Institute of Geriatric Medicine, Chinese Academy of Medical Sciences, Beijing Hospital, National Center of Gerontology of National Health Commission, 100730 Beijing, China; 2 Department of Laboratory Medicine, Beijing Hospital, National Center of Gerontology, Institute of Geriatric Medicine, Chinese Academy of Medical Sciences, 100730 Beijing, China; 3 Department of Geriatrics, Beijing Hospital, National Center of Gerontology, Institute of Geriatric Medicine, National Clinical Research Center for Geriatrics, Chinese Academy of Medical Sciences, 100730 Beijing, China

**Keywords:** Malnutrition, Aromatic amino acid, Biomarkers, Frailty, Metabolomics

## Abstract

**Background:**

Malnutrition is a common geriatric syndrome that is closely associated with adverse clinical outcomes and poses significant harm to older adults. Early assessment of nutritional status plays a crucial role in preventing and intervening in cases of malnutrition. However, there is currently a lack of measurable methods and biomarkers to evaluate malnutrition in older adults accurately. The aim of this study is to investigate the independent correlation between serum levels of amino acids and malnutrition in older adults, and to identify effective metabolomics biomarkers that can aid in the early detection of geriatric malnutrition.

**Methods:**

A total of 254 geriatric medical examination participants from Beijing Hospital were included in the study, consisting of 182 individuals with normal nutritional status (Normal group) and 72 patients at risk of malnutrition or already malnourished (MN group). Malnutrition was assessed using the Mini-Nutritional Assessment Short-Form (MNA-SF). Demographic data were collected, and muscle-related and lipid indexes were determined. Serum amino acid concentrations were measured using isotope dilution liquid chromatography-tandem mass spectrometry (LC-MS/MS). The correlation between serum amino acid levels and malnutrition was analyzed using non-parametric tests, partial correlation analysis, linear regression, and logistic regression.

**Results:**

The geriatric MN group exhibited significantly lower serum aromatic amino acid levels (*P* < 0.05) compared to the normal group. A positive correlation was observed between serum aromatic amino acid levels and the MNA-SF score (*P* = 0.002), as well as with known biomarkers of malnutrition such as body mass index (BMI) (*P* < 0.001) and hemoglobin (HGB) (*P* = 0.005). Multivariable logistic or linear regression analyses showed that aromatic amino acid levels were negatively correlated with MN and positively correlated with the MNA-SF score, after adjusting for some confounding factors, such as age, gender, BMI, smoking status, history of dyslipidemia, diabetes mellitus and frailty. Stratified analyses revealed that these trends were more pronounced in individuals without a history of frailty compared to those with a history of frailty, and there was an interaction between aromatic amino acid levels and frailty history (*P* = 0.004).

**Conclusion:**

Our study suggests that serum aromatic amino acids are independently associated with malnutrition in older adults. These results have important implications for identifying potential biomarkers to predict geriatric malnutrition or monitor its progression and severity, as malnutrition can result in poor clinical outcomes.

**Supplementary Information:**

The online version contains supplementary material available at 10.1186/s12877-024-04937-y.

## Introduction

Malnutrition among older adults has become a pressing global health issue, with far-reaching implications for individuals, families, and healthcare systems [1, 2], such as a higher incidence of complications and increased healthcare expenses for hospitalized patients [3]. Approximately 20 million community-dwelling older adults suffer from malnutrition, with a prevalence rate of 12.6% in China [4]. The problem is even more severe among hospitalized older patients, with malnutrition prevalence in Western countries estimated to be between 30 and 50%, and as high as 85% in long-term care facilities [3]. The causes of nutritional deficiency in older adults are multifaceted and their pathogenesis is intricate, involving various factors such as dietary intake, absorption, digestion, and metabolism [5, 6]. Treating malnutrition equally poses significant challenges, early identification, multimodal interventions, and personalized treatment based on the living environment and physical condition of older adults are required [1, 7]. Therefore, it is crucial to proactively recognize, evaluate, and prevent malnutrition in older adults at an early stage.

There is currently no precise definition of malnutrition in older adults. Nevertheless, the Global Leadership Initiative on Malnutrition has established criteria to identify malnutrition based on weight loss, loss of muscle mass, loss of body mass index (BMI), reduced food intake and absorption, and inflammation or disease burden [8, 9]. Screening tools for malnutrition in older adults should prioritize the most significant risk factors. The Mini-Nutritional Assessment (MNA) is the most commonly used tool in both long-term and short-term formats [10, 11]. Other screening tools for the assessment of malnutrition include the Subjective General Assessment [12] and the Nutritional Risk Screening 2002 [11]. However, the MNA is considered more appropriate for the nutritional assessment of elderly surgical patients compared to the Nutritional Risk Screening [11], which has been associated with a higher risk of ‘overdiagnosis’ of malnutrition in older adults [10, 13]. The MNA involves subjective assessments of lifestyle, health, and nutritional self-perceptions, which may introduce bias. Therefore, it is necessary to have objective and measurable methods in clinical practice to accurately evaluate malnutrition in older adults.

In recent years, laboratory biomarkers have emerged as potential tools for diagnosing malnutrition, assessing nutritional risk, and monitoring interventions. Several studies have identified BMI, hemoglobin (HGB), total cholesterol (TC), albumin (ALB), and serum leptin as biomarkers associated with malnutrition in older individuals [13–17]. While BMI is commonly used in clinical practice due to its convenience, it lacks well-established thresholds [15]. ALB is frequently used to diagnose malnutrition, but there is a lack of evidence-based clinical guidelines supporting its application in specific conditions and settings [15, 18]. Furthermore, albumin can only effectively identify severely malnourished patients, making it susceptible to underdiagnosis in practice. Similarly, HGB, TC, and total protein may also contribute to underdiagnosis of malnutrition due to the absence of appropriate cutoff values, thus making it difficult to accurately identify high-risk patients [15, 16]. It is worth noting that studies reporting malnutrition-related decreases in serum leptin concentration primarily focused on women, and may not be fully representative of the entire elderly population [17].

Recent advances in metabolomics technology have the potential to provide new tools for assessing malnutrition in older adults. A study discovered that seven branched-chain amino acids and related metabolites are significantly associated with thigh muscle cross-sectional area and fat-free mass index in older adults, and can potentially serve as serum markers for sarcopenia [19]. Another study examining biomarkers associated with sarcopenia and frailty in older adults found elevated levels of aspartate, asparagine, citrulline, ethanolamine, glutamate, sarcosine and taurine in older adults with sarcopenia and frailty, as along with higher levels of alpha-aminobutyric acid and methionine in controls [20]. Considering that malnutrition is a common predisposing factor for frailty and sarcopenia, the aforementioned findings may have a close relationship with geriatric malnutrition [21, 22].

With this in mind, our study focused on targeted amino acid profile metabolomics in order to explore the correlation between amino acids and malnutrition as a means of identifying new biomarkers for geriatric malnutrition. Amino acids are known to be crucial components for protein synthesis and metabolism. Clinical malnutrition arises from an imbalance between protein and energy intake and demand [23]. Furthermore, there is evidence suggesting that patients with trauma-induced malnutrition have lower levels of tryptophan and histidine [24]. However, there is still insufficient evidence linking amino acids to malnutrition in older individuals. Therefore, the objective of our study was to investigate the independent correlation between amino acids and geriatric malnutrition.

## Materials and methods

### Participants

This cross-sectional study recruited a total of 254 subjects (62–100 years old) who underwent physical examinations at the Geriatrics Medicine Department of Beijing Hospital from August 2016 to May 2018. The inclusion criteria required participants to be older than 60 years old, have the ability to comprehend and accomplish the questionnaire, and not have severe mental or cognitive disorders. Exclusion criteria included patients with malignant tumors, hematologic diseases, chronic obstructive pulmonary disease, autoimmune diseases, infectious diseases, and those taking amino acid supplements. The study was approved by the Medical Ethics Committee of Beijing Hospital of the National Health Commission, and all subjects provided informed consent.

Baseline demographics, such as age, gender, height, weight, blood pressure, smoking status, and medical history information (including diabetes, hyperlipidemia, frailty, and sarcopenia), were collected. After participants had fasted overnight, venous blood was taken in the morning, centrifuged to separate the serum, and stored at -80 ℃.

### Measurement of amino acid metabolites

A total of 10 serum amino acid metabolites, including tyrosine (Tyr), tryptophan (Trp), phenylalanine (Phe), valine (Val), leucine (Leu), isoleucine (Ile), glutamic acid (Glu), glycine (Gly), glutamine (Gln), and alanine (Ala), were determined by liquid chromatography-tandem mass spectrometry (LC-MS/MS) [25, 26]. For the analysis, 0.01 ml of mixed standards or serum samples was mixed with 0.01 ml of the isotope internal standards. Then, 1 ml of isopropanol was added and the mixture was vortexed, followed by centrifugation at 3500 rpm. Subsequently, 0.2 ml of the supernatant was pipetted into the injection vial, and eventually 3 ul of it was injected into a high performance liquid chromatography system equipped with an Agilent 1260 series (Santa Clara, CA, USA) and tandemly connected to an AB Sciex 5500 QTRAP mass spectrometer (Framingham, MA, USA) for analysis using LC-MS/MS.

### Assessment of malnutrition

Malnutrition was assessed using the MNA-SF. A score of ≥ 12 points was defined as the normal group, while scores < 7 and 8–11 were classified as the MN group.

### Measurement of other variables

The study utilized a fully automated flow cytometer to detect routine blood indicators such as red blood cells (RBC), white blood cells (WBC), platelets (PLT). Additionally, a Hitachi 7180 chemistry analyzer was used to measure routine biochemical parameters including hemoglobin (HGB), fasting blood glucose (FBG), total cholesterol (TC), triglycerides (TG), low-density lipoprotein cholesterol (LDL-C), high-density lipoprotein cholesterol (HDL-C), albumin (ALB), uric acid (UA), creatinine (CR), and blood urea nitrogen (BUN).

Appendicular skeletal muscle mass (ASM) was assessed using a bioelectrical impedance data acquisition system (Inbody720, BiospaceCo, Ltd, Seoul, Korea). The skeletal muscle mass index (SMI) was calculated as ASM/height squared (kg/m^2^), with a value lower than 7.0 kg/m^2^ indicating low SMI. Handgrip strength in both hands was measured twice using a digital dynamometer (WCS-II, Beijing, China) to assess low muscle strength, with a two-handed grip strength lower than 28 kg indicating low muscle mass. Gait speed was measured by averaging the results of two timed walking tests over a distance of more than 6 m, with a walking speed of less than 1.0 m/s indicating low gait speed.

### Statistic analysis

The data for continuous variables are typically presented as mean ± standard deviation for normally distributed data, and as median (P25, P75) for non-normally distributed data. Categorical variables are expressed as numbers (percentage). The normal distribution of the data was assessed using the Kolmogorov-Smirnov test. The comparison between the normal and MN groups was conducted using independent t-test and χ2 test, and a variance chi-square test was performed to compare whether the overall methods represented by multiple samples were equal before each analysis of t-test. Spearman’s correlation analysis was used to examine the association between amino acid concentrations and previously reported biomarkers, such as ALB, TC, HGB, and BMI or conventional risk factors, including MNA-SF score, age, BMI, systolic blood pressure (SBP), diastolic blood pressure (DBP), and clinical laboratory indicators. All statistical tests were two-tailed, and *P* values < 0.05 were considered statistically significant. The correlations between the continuous variable MNA-SF score and the levels of Tyr, Trp, Phe, and total aromatic amino acids (AAA) were analyzed using linear regression. The correlations between the dichotomous variable MN and the levels of Tyr, Trp, Phe, and AAA were analyzed using binary logistic regression, and further analyzed by stratifying them based on history of frailty and sarcopenia.

## Results

### Clinical characteristics of the study participants

The physical examination population consisted of 182 (71.7%) normal individuals and 72 (28.3%) patients with MN. Among the MN group, 46.6% exhibited reduced muscle mass index, 47.2% presented reduced muscle strength, and 38.8% had a low gait speed.

The clinical characteristics of all study participants are shown in Table 1. The MN group was significantly older (*P* < 0.001) compared to the normal group. Traditional risk factor indicators aligned with established studies, with the MN group having a lower BMI (*P* < 0.001), higher TC (*P* = 0.042), lower HGB (*P* = 0.002), and lower ALB (*P* = 0.060). Patients in the MN group had a higher prevalence of weakness and sarcopenia compared to the normal group. Muscle mass, SMI, and grip strength were significantly lower in the MN group compared to the normal group, while there was no significant difference in reduced gait speed between the two groups. Erythrocyte count, hemoglobin content, and TG were lower in MN subjects compared to normal subjects, and HDL-C was higher. Other biochemical parameters did not show significant differences between the two groups.

The levels of Tyr, Trp, Phe, and AAA were significantly lower in the MN group than those in the normal group. Among branched-chain amino acids (BCAA), Val, Ile, and total BCAA levels were also significantly lower in the MN group, and Ala followed the same trend. Glu, Gln, whereas Gly did not show any significant differences between the two groups.

**Table 1:** Clinical characteristics of the study participants with and without malnutrition

Variables	Normal (*n* = 182)	MN (*n* = 72)	P-value
**General information**			
Age, years	78.2 ± 7.5	82.0 ± 8.5	**< 0.001**
Male, n (%)	171 (94.5)	64 (88.9)	0.120
BMI, kg/m^2^	25.5 ± 3.4	23.2 ± 3.4	**< 0.001**
SBP, mmHg	129.2 ± 17.3	128.1 ± 15.9	0.663
DBP, mmHg	77.6 ± 56.1	70.5 ± 9.2	0.302
Diabetes, n (%)	61 (33.5)	24 (33.3)	0.978
Dyslipidemia, n (%)	111 (64.9)	46 (69.7)	0.486
Frailty, n (%)	102 (56.4)	54 (75.0)	**0.006**
Sarcopenia, n (%)	45 (27.8)	27 (46.6)	**0.009**
**Muscle-related parameters**			
ASM, kg	21.2 (19.0 ~ 23.0)	18.8 (17.3 ~ 21.6)	**< 0.001**
SMI, kg/m^2^	7.3 (6.8 ~ 7.9)	7.0 (6.4 ~ 7.3)	**< 0.001**
Low SMI	45 (27.8)	27 (46.6)	**0.009**
Handgrip strength, kg	31.2 (25.9 ~ 35.3)	28.1 (20.9 ~ 32.5)	**0.006**
Low muscle strength	45 (31.0)	25 (47.2)	**0.036**
Gait speed (m/s)	0.9 (0.7 ~ 1.1)	1.0 (0.7 ~ 1.0)	0.471
Low gait speed	82 (46.3)	26 (38.8)	0.292
**Clinical laboratory parameters**			
RBC, 10^12^/L	4.7 (4.4 ~ 5.0)	4.5 (4.2 ~ 4.7)	**0.010**
WBC, 10^9^ /L	5.9 (4.8 ~ 7.0)	5.7 (4.8 ~ 7.0)	0.958
PLT, 10^9^ /L	185.0 (158.0 ~ 214.0)	187.0 (170.0 ~ 224.8)	0.246
HGB, g/L	144.0 (134.0 ~ 150.0)	135.5 (130.0 ~ 145.5)	**0.002**
FBG, mmol/L	5.7 (5.3 ~ 6.4)	5.6 (5.2 ~ 6.2)	0.139
TG, mmol/L	1.3 (0.9 ~ 1.8)	1.1 (0.7 ~ 1.5)	**0.001**
TC, mmol/L	4.1 ± 1.0	4.4 ± 1.1	**0.042**
LDL-C, mmol/L	2.3 (1.8 ~ 2.9)	2.5 (1.8 ~ 3.0)	0.299
HDL-C, mmol/L	1.2 (1.0 ~ 1.4)	1.3 (1.1 ~ 1.5)	**0.031**
ALB, g/L	43.0 (41.0 ~ 44.0)	42.0 (41.0 ~ 43.0)	0.060
UA, µmol/L	347.5 (300.8 ~ 413.5)	346.0 (293.5 ~ 413.5)	0.842
CR, µmol/L	77.0 (66.0 ~ 91.0)	78.0 (70.0 ~ 94.3)	0.245
BUN, mmol/L	5.6 (4.5 ~ 6.8)	6.0 (4.8 ~ 6.9)	0.146
**Serum amino acids**			
Tyr, umol/L	16.3 (14.3 ~ 18.2)	15.2 (12.5 ~ 16.5)	**0.002**
Trp, umol/L	17.4(15.3 ~ 19.4)	16.0(14.3 ~ 17.7)	**0.002**
Phe, umol/L	19.7(17.3 ~ 22.5)	18.4(15.8 ~ 20.7)	**0.012**
AAA, umol/L	53.5 (48.2 ~ 59.1)	50.0 (43.6 ~ 55.0)	**0.002**
Val, umol/L	40.0(35.1 ~ 43.7)	37.6(33.7 ~ 42.4)	**0.037**
Leu, umol/L	25.4(22.5 ~ 29.1)	25.6(21.8 ~ 27.1)	0.088
Ile, umol/L	12.4(11.2 ~ 14.5)	11.6(10.2 ~ 13.7)	**0.015**
BCAA, umol/L	77.8(70.1 ~ 86.9)	74.3(65.9 ~ 82.5)	**0.030**
Gln, umol/L	26.5(21.6 ~ 30.5)	25.2 (19.9 ~ 31.2)	0.304
Glu, umol/L	22.1 (18.7 ~ 25.0)	22.1(19.2 ~ 24.3)	0.955
Gly, umol/L	39.0(33.7 ~ 48.3)	40.8(36.0 ~ 51.3)	0.215
Ala, umol/L	51.8(42.7 ~ 58.2)	44.9(40.3 ~ 53.7)	**0.003**

*Abbreviations*BMI, body mass index; SBP, systolic blood pressure; DBP, diastolic blood pressure; ASM, appendicular skeletal muscle mass; SMI, skeletal muscle index; RBC, red blood cells; WBC, white blood cells; PLT, platelets; HGB, hemoglobin; FBG, fasting glucose; TG, triglyceride; TC, total cholesterol; LDL-C, low-density lipoprotein cholesterol; HDL-C, high-density lipoprotein cholesterol; ALB, albumin; UA, uric acid; CR, creatinine; BUN, blood urea nitrogen; Tyr, tyrosine; Trp, tryptophan; Phe, phenylalanine; AAA, aromatic amino acid; Val, valine; Leu, leucine; Ile, isoleucine; BCAA, branched-chain amino acid; Gln, glutamine; Glu, glutamic acid; Gly, glycine; Ala, alanine.

Malnutrition was evaluated using the Mini-Nutritional Assessment Short-Form (MNA-SF).

Older adults were considered to be frail if they met three or more of these five criteria, prefrail if they met one or two criteria, and non-frailty if they met none of these criteria. A combined group of prefrail together with frailty was analyzed as the “frailty” group(non-frailty was the reference group) because of the small sample size of the frail group, according to the previous study".

We defined sarcopenia using the diagnostic algorithm of the Asian Working Group on Sarcopenia (AWGS) according to the presence of both low muscle mass and low muscle function (slow walking speed or low grip strength).

Data are mean ± SD, median (interquartile range) for continuous variables, or numbers (percentage) for categorical variables.

### Spearman correlation between amino acids and previously reported biomarkers or conventional malnutrition risk factors

**Fig. 1:**
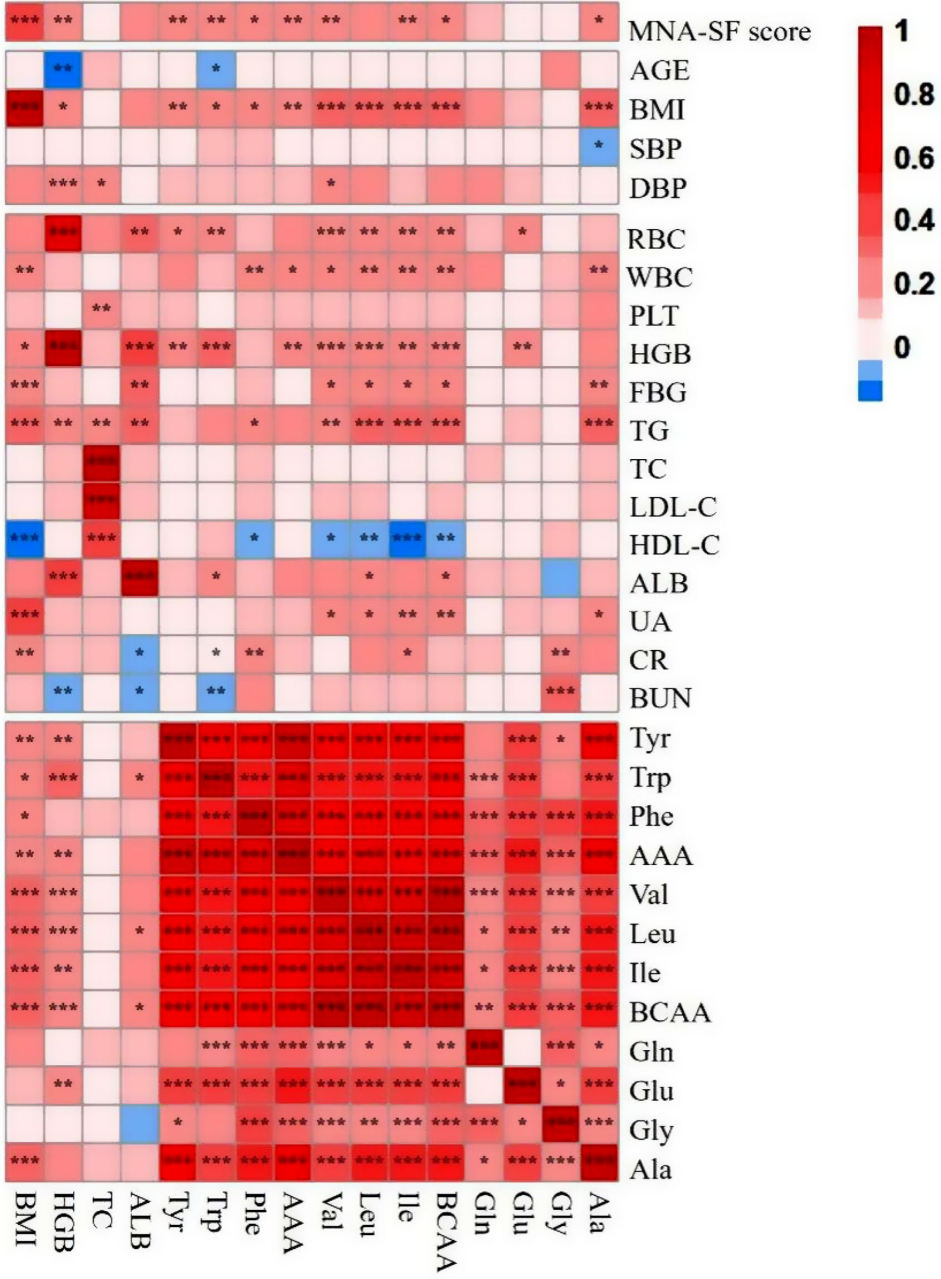
Spearman correlation (r) of AAA and previously reported biomarkers with other risk factors. *Abbreviations* MNA-SF score, Mini Nutrition Assessment-Short Form; BMI, body mass index; SBP, systolic blood pressure; DBP, diastolic blood pressure; RBC, red blood cells; WBC, white blood cells; PLT, platelets; HGB, hemoglobin; FBG, fasting glucose; TG, triglyceride; TC, total cholesterol; LDL-C, low-density lipoprotein cholesterol; HDL-C, high-density lipoprotein cholesterol; ALB, albumin; UA, uric acid; CR, creatinine; BUN, blood urea nitrogen; Tyr, tyrosine; Trp, tryptophan; Phe, phenylalanine; AAA, aromatic amino acid; Val, valine; Leu, leucine; Ile, isoleucine; BCAA, branched-chain amino acid; Gln, glutamine; Glu, glutamic acid; Gly, glycine; Ala, alanine. *, *P* < 0.05; **,*P* < 0.01; ***, *P* < 0.001

The results of the correlation analysis between known biomarkers, the MNA-SF score, conventional risk factors, and various amino acids are presented in Fig. 1. Tyr, Trp, and Phe, along with total AAA and BCAA except Leu and Ala, demonstrated a significant positive correlation with the MNA-SF score. AAA exhibited significant positive correlations with BMI, WBC, and HGB. Among the four reported biomarkers of malnutrition, BMI and HGB displayed a positive correlation with the MNA-SF score, while TC and ALB did not exhibit a significant correlation. The levels of Tyr, Trp, Phe, AAA, Val, Leu, Ile, total BCAA, and ALA showed significant positive correlations with BMI. Tyr, Trp, total AAA, BCAA, and Glu showed significant positive correlations with HGB. No significant correlation was observed between TC and amino acids. ALB displayed a significant positive correlation with Trp, Leu, and total BCAA.

### Regression analysis for serum AAA with malnutrition and MNA-SF score

**Fig. 2:**
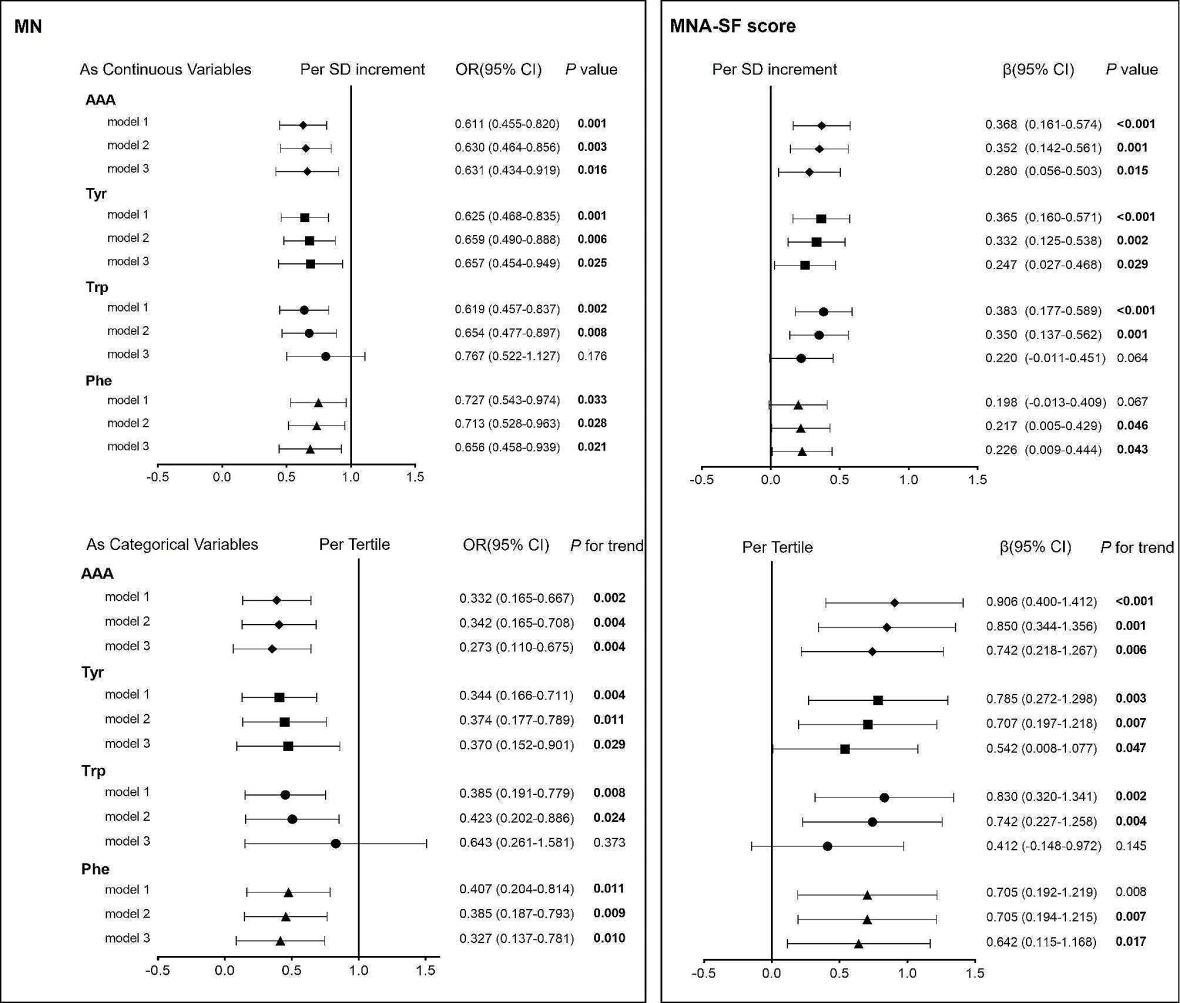
Odds ratios or β for MN and MNA-SF score according to AAA levels.Model 1: Crude risk. Model 2: Adjusted for age and gender. Model 3: Further adjusted for smoking status, SBP, DBP, BMI, diabetes, dyslipidemia, and frailty. Malnutrition risk was evaluated using the Mini-Nutritional Assessment Short-Form (MNA-SF)

As demonstrated in Fig. 2, multivariable logistic and linear regression were employed to examine the association between Tyr, Trp, Phe, total AAA levels and the MN and the MNA-SF score. When considering AAA as continuous variables, the levels of AAA, Tyr, Trp and Phe were found to be associated with a decreased prevalence of MN (OR < 1). Conversely, the MNA-SF score showed an increase with rising levels of these amino acids, except for elevated levels of phenylalanine. Upon adjusting for age and gender (model 2), it was observed that Tyr, Trp, Phe, and AAA levels were all significantly negatively correlated with MN, while the MNA-SF score was significantly positively correlated with them. Further adjustment for smoking status, SBP, DBP, BMI, medical history of diabetes mellitus, dyslipidemia, and frailty (model 3) revealed that Tyr, Phe, and AAA levels maintained a significant correlation with MN and the MNA-SF score, whereas Trp did not exhibit a significant correlation. For each increase of one standard deviation of AAA, the odds for MN decreased by 38.9% (OR = 0.611, 95% CI: 0.455-0.820; *P* = 0.001), while the MNA-SF score increased by approximately 37% (β = 0.368, 95% CI: 0.161–0.574, *P* < 0.001). When converting AAA from a continuous variable to a categorical variable based on tertiles, the results were remarkably similar to those obtained with continuous variables. In regression model 3, after adjusting for multivariate variables, individuals with T2 and T3 total AAA exhibited 50.3% and 72.7% lower odds of MN, respectively, compared with those with the lowest T1 of total AAA levels, whereas the MNA-SF score increased by 35.9% and 74.2%, respectively, showing a dose-response relationship (*P* trend < 0.01).

### Stratified analysis of the associations between AAA with malnutrition and MNA-SF score

The association between serum levels of each AAA and malnutrition was further stratified based on the presence or absence of a history of frailty and sarcopenia. The results of the interaction analysis with a history of frailty are presented in Table 2. After adjusting for age, gender, BMI, smoking status, history of dyslipidemia, and history of diabetes mellitus, Tyr, Trp, Phe, and total AAA were found to be negatively associated with MN in the group with no history of frailty. Moreover, the MNA-SF score significantly increased with higher levels of each aromatic amino acid. For each increase of one standard deviation of total AAA, the odds for MN decreased by 84.4% (OR = 0.156, 95% CI: 0.046-0.521; *P* = 0.003), while the MNA-SF score increased by approximately 62% (β = 0.616, 95% CI: 0.206–1.206, *P* < 0.001). However, in subjects with a history of frailty, there was no significant correlation between MN, the MNA-SF score, and AAA. Notably, there was an interaction between AAA, Trp, Phe, and a history of frailty illness (*P* interaction < 0.05). When stratified by medical history of sarcopenia, no statistically interaction was observed (*P* interaction > 0.05) as shown in Supplementary Table S1.

**Table 2:** Stratified analysis between AAA and MN, MNA-SF score according to Frailty

	MN			MNA-SF score		
	OR (95% CI)	*P*	*P*_interaction_	β (95% CI)	*P*	*P interaction*
**AAA**						
Yes	0.794 (0.514–1.226)	0.297	**0.004**	0.141 (-0.131-0.414)	0.359	**0.048**
No	0.156 (0.046–0.521)	**0.003**		0.616 (0.206–1.026)	**0.004**	
**Tyr**						
Yes	0.763 (0.494–1.179)	0.223	0.121	0.172 (-0.103-0.446)	0.221	0.311
No	0.370 (0.158–0.863)	**0.021**		0.408 (0.015–0.801)	**0.043**	
**Trp**						
Yes	1.027 (0.658–1.603)	0.906	**0.001**	0.049 (-0.230-0.327)	0.731	**0.015**
No	0.130 (0.032–0.531)	**0.005**		0.647 (0.223–1.070)	**0.003**	
**Phe**						
Yes	0.759 (0.502–1.146)	0.190	**0.015**	0.121 (-0.141-0.383)	0.366	0.113
No	0.187 (0.057–0.620)	**0.006**		0.499 (0.084–0.913)	**0.019**	

Values are adjusted for age, gender, smoking status, SBP, DBP, BMI, diabetes, and dyslipidemia

## Discussion

To the best of our knowledge, this study is the first to demonstrate the independent correlation between serum levels of aromatic amino acids and malnutrition in older adults. In this study, we analyzed the relationship of serum amino acid profiles, measured by targeted metabolomics, with the prevalence of malnutrition, as well as the MNA-SF score. Our findings indicate that geriatric patients with malnutrition had significantly lower levels of aromatic amino acids compared to normal older adults. These levels were also strongly associated with previously reported biomarkers for geriatric malnutrition. We also observed a negative correlation of serum levels of aromatic amino acids with the prevalence of malnutrition, as well as a positive correlation with the MNA-SF score in a dose-response manner. Importantly, these relationships are independent of age, gender, BMI, smoking status, dyslipidemia, diabetes and frailty. All the above significant results hint that AAA may be potential candidate biomarkers for early detection and prediction of geriatric malnutrition, as well as monitoring its severity or progression in the future. Quantitative detection of these biomarkers can reduce subjective bias, may be more sensitive to changes in the progression or severity of malnutrition, and may suggest possible intervention options through metabolic pathway information, compared to conventional malnutrition assessment scales.

At present, malnutrition is mainly assessed through various malnutrition screening tools [25], but these tools often yield inconsistent results and rely mostly on weight and some subjective recalls, which are prone to subjective bias [26]. Body weight is commonly used as an indicator to objectively assess malnutrition in older adults [27]. Our study aligns with this, showing that malnourished older adults tend to have a notably lower BMI compared to those classified as normal. Additionally, other reported biomarkers of malnutrition, including HGB and TC, were also confirmed in our study, showing significant differences between the MN group and normal older adults. Research suggests that serum ALB is an effective indicator of nutritional status in clinically stable conditions [28] and is commonly used in clinical settings. However, there was no notable difference in ALB levels between the two groups in our study. Although the aforementioned biomarkers are frequently used in clinical practice to screen for malnutrition, there is currently no universally accepted standard for identifying or diagnosing malnutrition.

Targeted amino acid metabolomics may provide new insights for identifying and diagnosing malnutrition among older adults. Previous research has shown that essential amino acid and histidine levels are generally low in elderly trauma patients and that tryptophan and histidine are significantly correlated with serum albumin [24]. We first analyzed the baseline amino acid profiles of the MN and Normal groups based on their MNA-SF score. The MN group exhibited notably reduced levels of AAA, BCAA and Ala, which were significantly and positively correlated with the MNA-SF score. There were no significant differences in Gly, Glu, and Gln levels between the two groups. Regression analyses revealed that lower AAA levels were associated with higher odds of malnutrition phenotype and lower MNA-SF scores, even after adjusting for age, smoking status, and past medical history. However, BCAA and Ala did not show this independent correlation. This specific amino acid profile may be linked to the nutritional and metabolic turnover characteristic of older adults.

Malnutrition, also known as protein-energy malnutrition, is primarily caused by a disparity between intake and requirement, resulting in inadequate protein and energy intake [29, 30]. Therefore, ensuring adequate energy and protein intake is essential for preventing malnutrition in older individuals. Protein requirements are higher in older adults due to their catabolic metabolism triggered by underlying diseases [23, 31]. Phe and Tyr are indispensable for protein synthesis, and Phe is converted intracellularly to Tyr by the enzyme phenylalanine hydroxylase, which is a precursor of the neurotransmitters dopamine, norepinephrine, and epinephrine [32]. A study on the collagen amino acid composition of rat liver and the effects of initial protein biosynthesis found that hepatic free amino acid pools doubled under tryptophan loading [33]. AAA have also been identified as potential biomarkers linked to insulin resistance and may have a positive impact on improving insulin resistance [34]. Phe and the metabolite n-hydroxyphenylacetic acid could potentially aid in enhancing the host’s metabolism [35]. These above reports demonstrated the importance of AAA in maintaining nutritional and metabolic homeostasis. The findings of this study highlighted a deficiency in AAA among malnourished older adults, offering valuable insights for future interventions aimed at malnourished and at-high risk populations.

Compared to the 12.6% prevalence of malnutrition in the China Health and Retirement Longitudinal Study [4], our study found that only 1.6% of the physical examination population was malnourished, while 26.8% were at risk of malnutrition. This difference may be attributed to the characteristics of the study populations. Our participants were elderly individuals seeking medical consultations at hospital outpatient clinics, whereas a significant portion of the previous study’s population was from rural areas [4]. Economic disparities and variances in health-seeking behaviors could explain the discrepancy in malnutrition rates between the studies. Unlike previous research that relied on BMI alone, our study utilized the MNA-SF score scale for a more comprehensive assessment of malnutrition [11]. By combining the malnourished and at high risk groups for analysis, we suggest that AAA could serve as a marker for early recognition of malnutrition.

Malnutrition is a significant factor contributing to both frailty and sarcopenia [20]. Weight loss in older adults is often accompanied by muscle mass loss, resulting in reduced strength, mobility, and immune function, which are all characteristic of frailty. The presence of malnutrition in older adults can elevate the risk of hospitalization, functional dependence, and mortality within the frail population [36]. Therefore, we further explored whether interactions exist between AAA levels and these two potential effect modifiers, i.e. frailty and sarcopenia. The relationship between AAA levels and malnutrition was more prominent in individuals without a history of frailty compared to those with a history of frailty, and there was an interaction between AAA levels and frailty history, aiding personalized future malnutrition management. The lack of statistical significance in the frailty group may be attributed to the small sample size post subgroup classification, highlighting the need for a larger sample size to validate this relationship in future studies. Furthermore, the presence of other diseases accompanying frailty or factors influencing the association between AAA and malnutrition cannot be ruled out. In contrast, no significant interaction were observed between AAA levels and sarcopenia history. In general, it will be essential to investigate this specific role of AAA in malnutrition in a larger sample size of the frailty or sarcopenia population while controlling for potential confounding factors.

This study has some limitations. Firstly, the sample size was small (*n* = 254), and further validation with a larger sample size is required. Secondly, the study did not differentiate between individuals at risk of malnutrition and those with malnutrition but defined anyone with an MNA-SF score of lower than 12 as MN, resulting in the inclusion of both groups and the number of patients with malnutrition was higher than the actual number of patients. Lastly, the present study was cross-sectional and could not determine a causal relationship between AAA levels and malnutrition, further prospective studies and research on related mechanisms are needed to verify this relationship.

## Conclusion

Our study suggests that serum aromatic amino acids are independently associated with malnutrition in older adults. These findings have important implications for identifying potential biomarkers to predict geriatric malnutrition or monitor its progression and severity, as malnutrition can result in poor clinical outcomes.

### Electronic supplementary material

Below is the link to the electronic supplementary material.

Supplementary Material 1

## Data Availability

The datasets used and/or analysed during the current study are available from the corresponding author on reasonable request.

## Abbreviations

MNA: Mini Nutrition Assessment
MNA-SF: Mini Nutrition Assessment-Short Form
BMI: Body mass index
SBP: Systolic blood pressure
DBP: Diastolic blood pressure
RBC: Red blood cells
WBC: White blood cells
PLT: Platelets
HGB: Hemoglobin
FBG: Fasting glucose
TG: Triglyceride
TC: Total cholesterol
LDL-C: Low-density lipoprotein cholesterol
HDL-C: High-density lipoprotein cholesterol
ALB: Albumin
UA: Uric acid
CR: Creatinine
BUN: Blood urea nitrogen
Tyr: Tyrosine
Trp: Tryptophan
Phe: Phenylalanine
AAA: Aromatic amino acid
Val: Valine
Leu: Leucine
Ile: Isoleucine
BCAA: Branched-chain amino acid
Gln: Glutamine
Glu: Glutamic acid
Gly: Glycine
Ala: Alanine
ASM: Appendicular skeletal muscle mass
SMI: Skeletal muscle mass index
LC-MS/MS: Liquid chromatography-tandem mass spectrometry

## References

[1] Norman K, Haß U, Pirlich M (2021). Malnutrition in older adults-recent advances and remaining challenges. Nutrients.

[2] Corish CA, Bardon LA (2019). Malnutrition in older adults: screening and determinants. Proc Nutr Soc.

[3] Keller U (2019). Nutritional laboratory markers in Malnutrition. J Clin Med.

[4] Wei JM, Li S, Claytor L, Partridge J, Goates S (2018). Prevalence and predictors of malnutrition in elderly Chinese adults: results from the China Health and Retirement Longitudinal Study. Public Health Nutr.

[5] Serra-Prat M, Mans E, Palomera E, Clavé P (2013). Gastrointestinal peptides, gastrointestinal motility, and anorexia of aging in frail elderly persons. Neurogastroenterol Motil.

[6] Bauer JM, Haack A, Winning K, Wirth R, Fischer B, Uter W (2010). Impaired postprandial response of active ghrelin and prolonged suppression of hunger sensation in the elderly. J Gerontol Biol Sci Med Sci.

[7] Volkert D, Beck AM, Cederholm T, Cereda E, Cruz-Jentoft A, Goisser S (2019). Management of Malnutrition in older patients-current approaches, evidence and open questions. J Clin Med.

[8] Cederholm T, Jensen GL, Correia MITD, Gonzalez MC, Fukushima R, Higashiguchi T, GLIM Core Leadership Committee; GLIM Working Group (2019). GLIM criteria for the diagnosis of malnutrition - A consensus report from the global clinical nutrition community. Clin Nutr.

[9] de van der Schueren MAE, Keller H, Cederholm T, Barazzoni R, Compher C, GLIM Consortium (2020). Global Leadership Initiative on Malnutrition (GLIM): Guidance on validation of the operational criteria for the diagnosis of protein-energy malnutrition in adults. Clin Nutr.

[10] Cereda E (2012). Mini nutritional assessment. Curr Opin Clin Nutr Metab Care.

[11] Zhou J, Wang M, Wang H, Chi Q (2015). Comparison of two nutrition assessment tools in surgical elderly inpatients in Northern China. Nutr J.

[12] Detsky AS, Baker JP, Mendelson RA, Wolman SL, Wesson DE, Jeejeebhoy KN (1984). Evaluating the accuracy of nutritional assessment techniques applied to hospitalized patients: methodology and comparisons. JPEN J Parenter Enter Nutr.

[13] Kuzuya M, Kanda S, Koike T, Suzuki Y, Satake S, Iguchi A (2005). Evaluation of Mini-nutritional Assessment for Japanese frail elderly. Nutrition.

[14] Cabrerizo S, Cuadras D, Gomez-Busto F, Artaza-Artabe I, Marín-Ciancas F, Malafarina V (2015). Serum albumin and health in older people: review and meta analysis. Maturitas.

[15] Zhang Z, Pereira SL, Luo M, Matheson EM (2017). Evaluation of blood biomarkers Associated with risk of Malnutrition in older adults: a systematic review and Meta-analysis. Nutrients.

[16] Kuzuya M, Izawa S, Enoki H, Okada K, Iguchi A (2007). Is serum albumin a good marker for malnutrition in the physically impaired elderly?. Clin Nutr.

[17] Amirkalali B, Sharifi F, Fakhrzadeh H, Mirarefein M, Ghaderpanahi M, Badamchizadeh Z (2010). Low serum leptin serves as a biomarker of malnutrition in elderly patients. Nutr Res.

[18] Raiten DJ, Namasté S, Brabin B, Combs G, L’Abbe MR, Wasantwisut E (2011). Executive summary–biomarkers of Nutrition for Development: building a Consensus. Am J Clin Nutr.

[19] Lustgarten MS, Price LL, Chale A, Phillips EM, Fielding RA (2014). Branched chain amino acids are associated with muscle mass in functionally limited older adults. J Gerontol Biol Sci Med Sci.

[20] Calvani R, Picca A, Marini F, Biancolillo A, Gervasoni J, Persichilli S (2018). A distinct pattern of circulating amino acids characterizes older persons with physical Frailty and Sarcopenia: results from the BIOSPHERE Study. Nutrients.

[21] Cruz-Jentoft AJ, Kiesswetter E, Drey M, Sieber CC (2017). Nutrition, frailty, and Sarcopenia. Aging Clin Exp Res.

[22] Calvani R, Miccheli A, Landi F, Bossola M, Cesari M, Leeuwenburgh C (2013). Current nutritional recommendations and novel dietary strategies to manage Sarcopenia. J Frailty Aging.

[23] Bauer J, Biolo G, Cederholm T, Cesari M, Cruz-Jentoft AJ, Morley JE (2013). Evidence-based recommendations for optimal dietary protein intake in older people: a position paper from the PROT-AGE Study Group. J Am Med Dir Assoc.

[24] Dawson B, Favaloro EJ (2009). High rate of deficiency in the amino acids tryptophan and histidine in people with wounds: implication for nutrient targeting in wound management–a pilot study. Adv Skin Wound Care.

[25] Power L, de van der Schueren MAE, Leij-Halfwerk S, Bauer J, Clarke M, Visser M (2019). Development and application of a scoring system to rate malnutrition screening tools used in older adults in community and healthcare settings - a MaNuEL study. Clin Nutr.

[26] Poulia KA, Yannakoulia M, Karageorgou D, Gamaletsou M, Panagiotakos DB, Sipsas NV (2012). Evaluation of the efficacy of six nutritional screening tools to predict malnutrition in the elderly. Clin Nutr.

[27] Cederholm T, Barazzoni R, Austin P, Ballmer P, Biolo G, Bischoff SC (2017). ESPEN guidelines on definitions and terminology of clinical nutrition. Clin Nutr.

[28] Campion EW, deLabry LO, Glynn RJ (1988). The effect of age on serum albumin in healthy males: report from the normative aging study. J Gerontol.

[29] Kinosian B, Jeejeebhoy KN (1995). What is malnutrition? Does it matter?. Nutrition.

[30] Omran ML, Morley JE (2000). Assessment of protein energy malnutrition in older persons, part II: Laboratory evaluation. Nutrition.

[31] Deutz NE, Bauer JM, Barazzoni R, Biolo G, Boirie Y, Bosy-Westphal A (2014). Protein intake and exercise for optimal muscle function with aging: recommendations from the ESPEN Expert Group. Clin Nutr.

[32] Flydal MI, Martinez A (2013). Phenylalanine hydroxylase: function, structure, and regulation. IUBMB Life.

[33] Pechenova TN, Sushkova VV, Solodova EV, Gulyĭ MF (1983). Vliianie Izbytka Triptofana v ratsione na aminokislotnyĭ sostav kollagena kozhi i nachal’nyĭ étap biosinteza belka v pecheni krys [Effect of tryptophan excess in a diet on amino acid composition of skin collagen and on an initial stage of protein biosynthesis in rat liver]. Ukr Biokhim Zh (1978).

[34] Zhou Y, Tang J, Du W, Zhang Y, Ye B-C (2024). Screening potential biomarkers associated with insulin resistance in high-fat diet-fed mice by integrating metagenomics and untargeted metabolomics. Microbiol Spectr.

[35] Jin Z, Yang Y, Cao Y, Wen Q, Xi Y, Cheng J (2023). The gut metabolite 3-hydroxyphenylacetic acid rejuvenates spermatogenic dysfunction in aged mice through GPX4-mediated ferroptosis. Microbiome.

[36] Moraes MB, Araujo CFM, Avgerinou C, Vidal EIO (2018). Nutritional interventions for the treatment of frailty in older adults: a systematic review protocol. Med (Baltim).

