# Supplementary material for: Targeted metabolomic profiles of serum amino acids are independently correlated with malnutrition in older adults
Source: BMC Geriatr. 2024 Apr 15;24:341. doi: 10.1186/s12877-024-04937-y (PMC11020810; doi:10.1186/s12877-024-04937-y)
Supplement: Supplementary file 1 — Supplementary Material 1 [file 12877_2024_4937_MOESM1_ESM.docx]

**Table S1. Stratified analysis between AAA and MN, MNA-SF score according to Sarcopenia**

|  | MN | | |
| --- | --- | --- | --- |
|  | OR (95% CI) | *P* | *P* _interaction_ |
| AAA |  |  |  |
| Yes | 0.686 (0.357-1.319) | 0.258 | 0.479 |
| No | 0.570 (0.358-0.908) | **0.018** |  |
| Tyr |  |  |  |
| Yes | 0.778 (0.425-1.425) | 0.416 | 0.280 |
| No | 0.567 (0.358-0.898) | **0.016** |  |
| Trp |  |  |  |
| Yes | 0.854 (0.406-1.798) | 0.678 | 0.340 |
| No | 0.621 (0.379-1.018) | 0.059 |  |
| Phe |  |  |  |
| Yes | 0.642 (0.342-1.206) | 0.168 | 0.992 |
| No | 0.670 (0.430-1.043) | 0.076 |  |

Values are adjusted for age, gender, smoking status, SBP, DBP, BMI, diabetes, and dyslipidemia.
